# Supplementary material for: Learning curve and surgical outcome of robotic assisted colorectal surgery with ERAS program
Source: Sci Rep. 2022 Nov 29;12:20566. doi: 10.1038/s41598-022-24665-w (PMC9709162; doi:10.1038/s41598-022-24665-w)
Supplement: Supplementary file 1 — Supplementary Information 1. [file 41598_2022_24665_MOESM1_ESM.zip › LSC Raw data-Ñ[▒K/146/O_ñw╝╨░O▒Kñσ.pdf]

正式摘要(電子簽章：0000002322143201)

請選擇...reporter

臺中榮民總醫院

Operation Note

[redacted]

- 手術日期：2021/12/16
- 手術醫師：蔣鋒帆，VS 邱騰逸，Fellow 林陳彥，R 蔡濬樟，R 陳怡豪
- Anesthesia：GE
- Position：Lithotomy
- Aseptic agents：Betadine Aqua，Zephnan

Diagnosis

- Pre-operative diagnosis : Diverticulosis and diverticulitis or Ca of S colon
- Post-operative diagnosis : Diverticulitis over S-colon s/p laparoscopic anterior resection

Surgical Indication

- For removing the suspicious, possibly diverticulitis involved S-colon

Operation

- 1. Laparoscopic anterior resection
  2. Intraoperative colonoscopy : (-)
  3. High ligation (+) : At IMA root
  4. EndoGIA (60-4.1) X1 + EEA (29 mm) were applied
  5. Proximal cut end: 3cm, distal cut end: > 10 cm
  6. No drainage tube was placed into the abdominal cavity
  7. The ADHESION BARRIER FILM was then placed below the para-umbilical extended wound.
  8. The colon was not taken down from the splenic flexure site

Finding

- 1. Severely adhesion was noted over the S-colon and the pelvic side wall, which was compatible with abd CT impression. The primary evaluation of the specimen revealed no obvious tumor like component.
  2. No other gross tumor nor polyps was found
  3. No obvious ascites was noted
  4. While performing the lysis between the S-colon and the pelvic side wall, a small site of bowel perforation was found. (iatrogenic). It was later removed with the specimen.
  5. Bilateral doughnuts : intact ; Air leak test: Done and no air leak was found

Procedure

- 1. General endotracheal anesthesia was induced and then the patient was placed in modified Trendelenburg lithotomy position. Preoperative antibiotics were given. A Foley catheter and a nasogastric tube were placed. The abdomen was prepped and draped in standard sterile fashion.
  2. A small vertical incision was made right next to the umbilicus and a 12 mm trocar was inserted into the peritoneal cavity by open method. Peumoperitoneum was established with CO2 inflation. Laparoscopy was inserted and then another 12 mm trocar was inserted over RLQ. Extra three 5 mm trochars were inserted over RUQ, LUQ and LLQ of abdomen. The operative findings were mentioned as above.
  3. The small bowel was inspected and retracted to the right by grasper. The sigmoid colon was then held by grasper and the peritoneum overlying the mesentery was then scored with electrocautery. The inferior mesentery artery was identified, ligated below the left colic artery with endoclips, and transected, so was inferior mesentery vein. Dissection was done above the Gerota's fascia and

- toward the peritoneal attachments.
  4. The arcade vessels to the sigmoid colon were also divided. Distal descending colon and upper rectum were mobilized. One linear staplers (EndoGIA 60-4.1) was then used to divide the rectum.
  5. Incision was further extended from the middle scope wound. The proximal colon was then taken out for carefully inspection, and to make sure it has an excellent blood supply. The mesenteric fat was gently cleared from the terminal 2-3 mm of the bowel ends.
  6. The field was isolated with moist pad and the bowel opened and sized. It was determined that a 29 mm circular stapler could be accommodated. A pursestring suture of 2-0 prolene was placed, the anvil was inserted, and then the pursestring suture was tied.
  7. The anal canal was irrigated with distil water and then the circular stapler was introduced through the anus and pierced through the mid-portion of the stapler line.
  8. The proximal bowel with anvil in place was engaged with the circular stapler and the stapler was then fired. Two complete donuts were obtained and the distal one was submitted to pathology for margin confirmation.
  9. The anastomosis was tested for leaks with irrigation solution and air introduced through the rectum and was found to be patent and intact.
  10. The possible bleeder was then checked thoroughly.
  11. The ADHESION BARRIER FILM was then placed below the central extended wound.
  12. The fascia of midline wound was closed with interrupted sutures of Num. 1 Vicryl. The fascia of the 12 mm trochar was also closed with 1-0 Vicryl.
  13. Skin were closed with interrupted 3-0 Nylon, and covered with Medipore.
  14. The patient tolerated the procedure well and was brought to the POR in stable condition.
- Estimated blood loss: minimum
- 器械數量: 正確
- 紗布數量: 正確

Image

- PAINT
  - abdomen

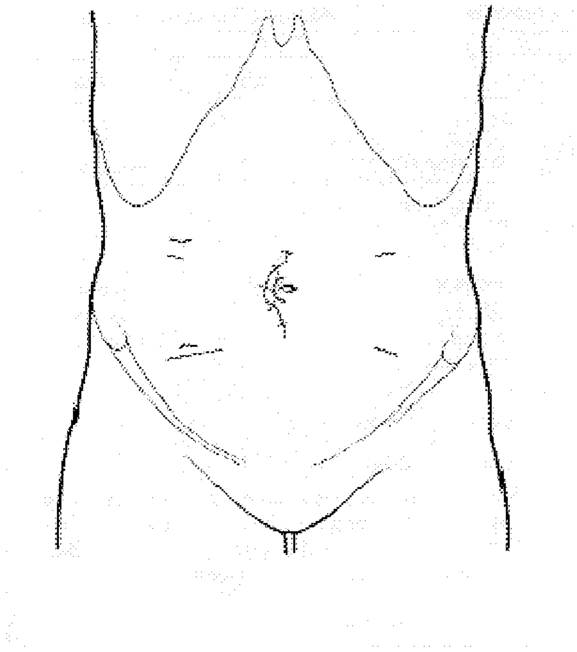

  - colon & rectum

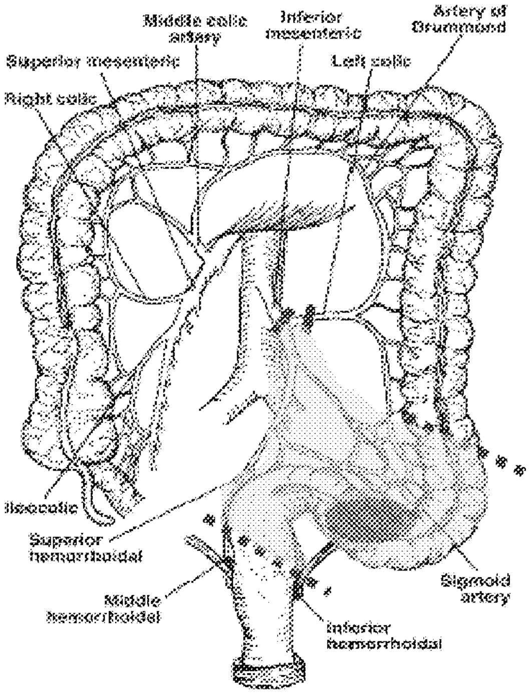

- The resected S-colon

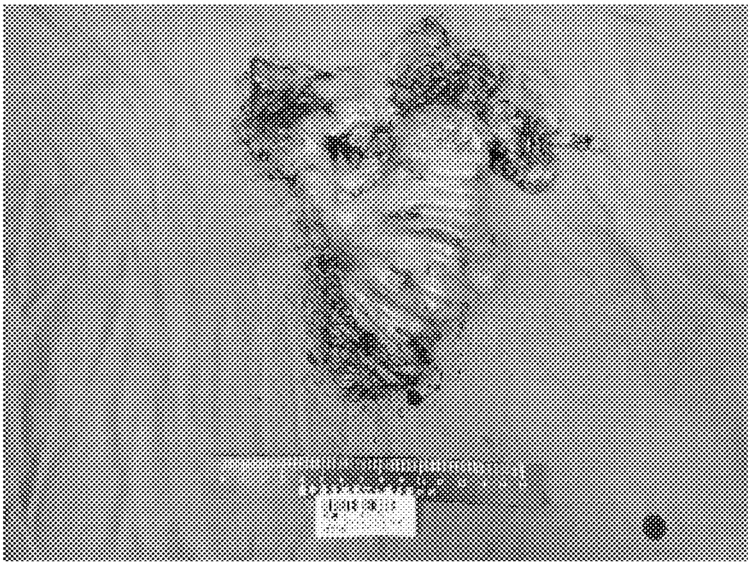

The severely inflammation pattern was noted

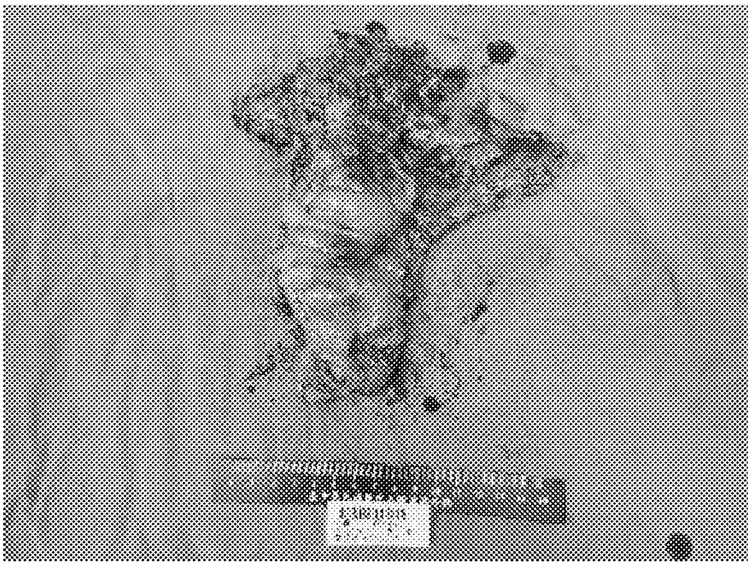

No obvious tumor like lesions were found

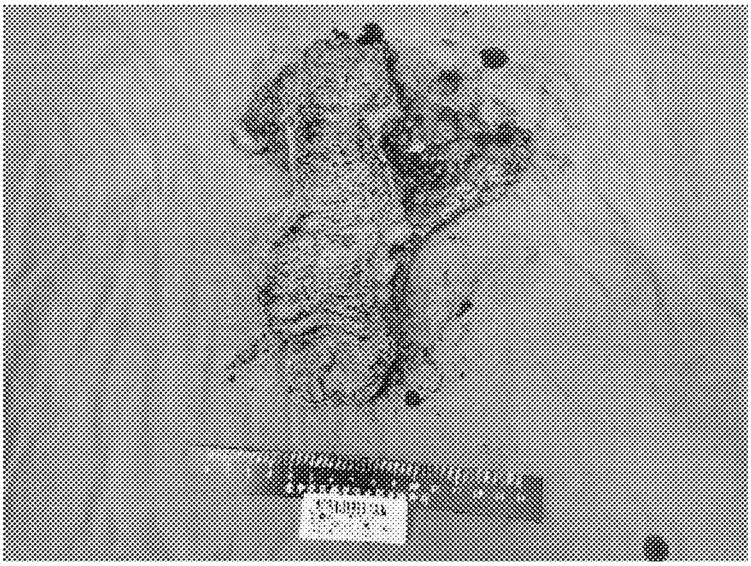

主任 2062F蔣鋒帆 / 醫師 2301H林陳彥

正式摘要**(**電子簽章：**000000232143201)**

請選擇...reporter

doctorRank:B
